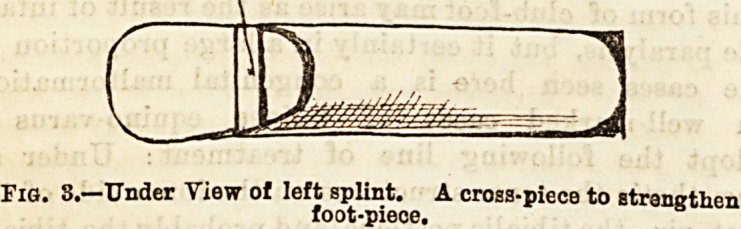# Treatment of Club-Foot

**Published:** 1893-06-10

**Authors:** W. M. Barlay

**Affiliations:** Surgeon to the Bristol General Hospital


### BRISTOL GENERAL HOSPITAL.

### TREATMENT OF CLUB-FOOT.

By W. M. BARLAY, F.R.C.S., Surgeon to the Bristol General Hospital.

Club-foot is one of the commonest deformities met with among children, and of its several varieties, talipes equinus, with more or less of varus combined with it, is certainly the one most frequently seen. This form of club-foot may arise as the result of infantile paralysis, but it certainly in a large proportion of the cases seen here is a congenital malformation. In well-marked cases of talipes equino-varus I adopt the following line of treatment: Under an anæsthetic the tense structures on the inner side of the foot, viz., the tibialis posticus (and probably the tibialis anticus as well), and the calcaneo-scaphoid ligament are divided by one subcutaneous incision as recommended by R. W. Parker. A curved tenotome is "entered immediately in front of the anterior border of the internal malleolus, the blade as far as possible being kept between the ligaments to be divided and the superjacent skin. The blade is then turned against the surface of the ligament, and by means of a gentle sawing action is made to divide it. As the superficial fibres are divided deeper ones come into play, and must in their turn be divided until bone is reached." There is often free oozing of blood from the puncture, and it may even be squirted out when the foot is manipulated, but it has never given any serious trouble. While this puncture is compressed by a pledget of lint the tendo Achillis is next divided in the ordinary way at a point about an inch above its insertion into the os calcis, the knife being entered at the inner side so as to avoid wounding the tibial artery. In one case in which this artery was presumably wounded the foot became blanched and cold, but although a good deal of œdema of the foot followed, which lasted a week or two, no permanent ill resulted. A pledget of carbolic gauze was bandaged over the puncture, and the foot put on a splint and surrounded by cotton wool. In carrying out these measures the whole foot is rendered aseptic with one in twenty carbolic lotion, and the punctures covered with carbolic gauze (Lister's new double-cyanide gauze would be equally efficacious, and is quite unirritating), and a little corrosive sublimate wood-wool, or boracic lint. The foot is then fixed in a plaster of Paris splint reaching up to the knee, being held forcibly in its rectified position till the plaster has set. A starched bandage would probably be quite as efficacious if materials for the other are not at hand. The toes are left in view so that any undue compression by the splint may be at once detected and removed. After a week or ten days, when the punctures will be sealed and the foot partially accustomed to its new position, the plaster splint is removed (soaking in warm water is the best way of doing this if the plaster is hard or thick), the leg

Fig. 1.—Side view of Left Splint.

Fig. 2.—The same, with loop of bandage in position.

Fig. 3.—Under View of left splint. A cross-piece to strengthen foot-piece.

washed and the following splint applied: A zinc or tin splint in the form of a trough fitting round the back and sides of the leg, but extending higher over the inner ankle (Fig. 1), with an opening opposite the heel, and a foot-piece. A loop of bandage or webbing is put up through the heel opening as in Fig. 2, the foot put through the loop, and the ends drawn down over the bend of the ankle, forcing the heel well down into the opening, and the sole as flat as possible against the foot-piece. The two ends are then turned up and crossed over the splint and foot, and the whole splint firmly bandaged on to the foot and leg. The splint is generally covered with wash-leather, and the foot and leg surrounded with strips of lint so as to prevent chafing. The advantage of substituting these splints for the plaster splint is that they can be removed and readjusted as often as necessary, and after a time, if the mother or nurse is intelligent, can be taken off every day by herself when the child is washed. The progress of the case can also be watched more easily.

In slighter cases of talipes these splints can be used as the sole means of treatment without any tenotomy. In either case, if carefully and intelligently carried out, and for a long enough time they give in the majority of cases very good results; but, like any other treatment, this method will fail if these points are not heedfully attended to.

## Figures and Tables

**Fig. 1. f1:**
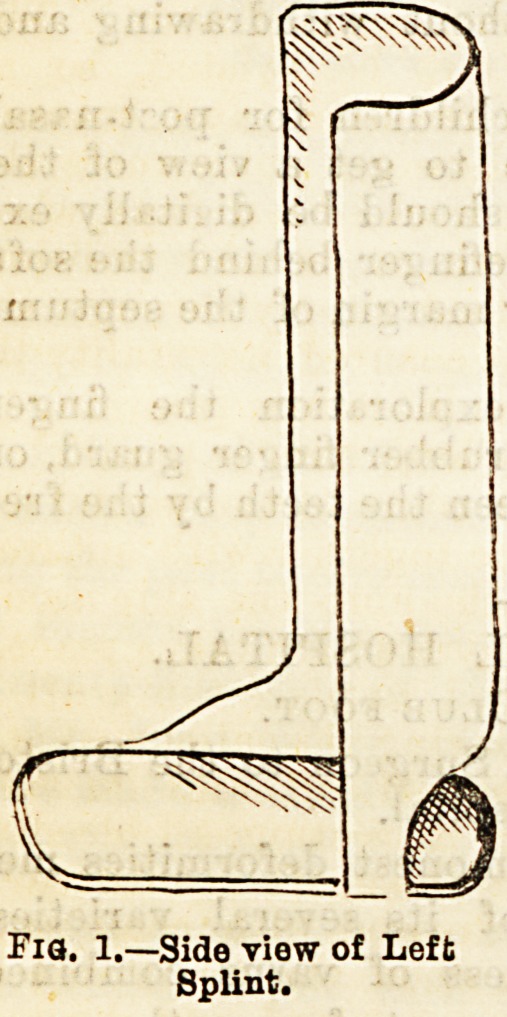


**Fig. 2. f2:**
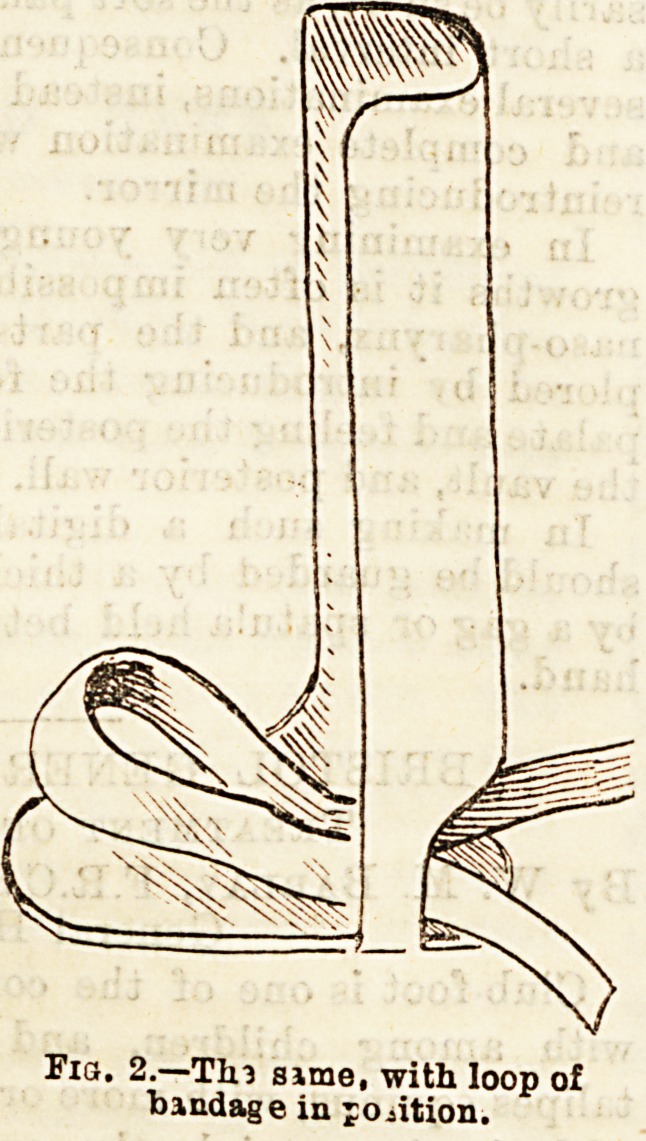


**Fig. 3. f3:**